# Transmission of Antibiotic Resistant Bacteria and Genes: Unveiling the Jigsaw Pieces of a One Health Problem

**DOI:** 10.3390/pathogens9060497

**Published:** 2020-06-22

**Authors:** Carla Novais, Ana R. Freitas

**Affiliations:** UCIBIO/REQUIMTE, Departamento de Ciências Biológicas, Laboratório de Microbiologia, Faculdade de Farmácia, Universidade do Porto, Rua Jorge Viterbo Ferreira, 228, 4050-313 Porto, Portugal

**Keywords:** transmission of bacterial pathogens, antibiotic resistance surveillance and epidemiology, virulence and biofilm production in antibiotic resistant bacteria, clones and mobile genetic elements promoting transmission of antibiotic resistance, genomics and bioinformatics analysis, genetics and evolution, infection prevention and control, One Health

## Abstract

Antimicrobial Resistance is one of the major Global Health challenges of the twenty-first century, and one of the World Health Organization’s (WHO) top ten global health threats. The evolution of antibiotic resistance among bacterial pathogens requires urgent concerted global efforts under a One Health approach integrating human, animal, and environmental surveillance data. This is crucial to develop efficient control strategies and counteract the spread of multidrug-resistant pathogens. The studies in this Special Issue have evidenced the hidden role of less common species, unusual clones or unexplored niches in the dissemination of antimicrobial resistance between different hosts. They reinforce the need for large-scale surveillance studies tracing and tracking both antibiotic resistance and metal tolerance in different bacterial species.

Antimicrobial resistance (AMR) is a well-reckoned major public health threat occurring at the animal–human–environment ecosystem interface that does not recognize geographical or host barriers. This complex and multifaceted problem poses a formidable challenge to antibiotic therapy, currently threatening the world to rewind the clock to the pre-antibiotic era [1]. Despite the increasing knowledge of microorganism communities’ composition and evolution in multiple ecosystems, it remains difficult to assess AMR dissemination and transmission between different hosts, as all species (microbiota) present in a given One Health microbiosphere (animals, food, sewage, soil, and aquatic environments) should be considered [2]. Different international entities (e.g., the World Health Organization (WHO) and the Food and Agriculture Organization of the United Nations) have been taking collective actions under a holistic One Health approach to minimize the emergence and spread of AMR. As addressed by the WHO, antimicrobials used to treat several infectious diseases in animals may be the same or similar to those used for humans, making the transmission and spread of AMR bacteria in humans inseparable from that occurring in animals and the environment [3]. Additionally, the increasing use of other antimicrobials (e.g., metals and disinfectants) in human, animal and environmental contexts is recognized as a potential driver for AMR selection. Thus, the spread of multiple and variable AMR bacteria, genes and mobile genetic elements across human, animal and environmental compartments is a complex process occurring through innumerable pathways (via wastewaters, soils, manure, direct contact between humans and animals, and food exposure) facilitating the continuous genetic exchange, recombination and evolution of bacteria and AMR traits [4].

To better understand the dynamics influencing the selection and transmission of AMR, this Special Issue aims to bring together research studies related to the transmission of AMR bacterial pathogens within and between different hosts and environments, as well as the impact of emergent clones and mobile genetic elements in AMR persistence and dissemination. The studies published in this research topic (four research articles and one review) have strengthened the emergence of less common species or unusual strains playing a role in the dissemination of AMR between different hosts. They also stress the importance of including unexplored non-clinical settings in large-scale surveillance studies tracking AMR resistance as well as other drivers with AMR impact, such as bacterial tolerance to metals.

Schell et al., detected invasive infection rates of 2:1 of *Enterococcus faecalis* and *Enterococcus faecium*, respectively, in a medium-sized Argentinian hospital and characterized their population structure [5]. Most of the *E. faecalis* belonging to diverse clonal lineages were resistant to high levels of resistance (HLR) to gentamicin and streptomycin, with a particular clone (ST9) that was also a producer of beta-lactamases (*bla^+^*) occurring in several hospital wards. This clone represents one of the few *bla^+^*-*E. faecalis* described to date with the majority being previously documented in late 1980s in Argentina, the USA, Canada and Lebanon. *E. faecalis* ST9 (*bla*+, HLR-gentamicin) suggests the presence of local hidden reservoirs for multidrug resistant *E. faecalis*. Additionally, this paper reported the occurrence of *E. faecium* clones carrying *vanA* or *vanB* genes belonging to clonal lineages (e.g., ST25, ST792) underrepresented in human infections in hospitals in western countries. These results reflect the relevance of local epidemiology and of the study of enterococci population structure in different locations to understand the emergence and spread of AMR.

Adesina et al., identified *Escherichia fergusonii* in septic wound samples obtained from patients at a general hospital in Lagos, Nigeria [6]. *E. fergusonii* is often identified as *Escherichia coli* or *Shigella* sp. due to the low discrimination power of phenotypic methods. However, a polyphasic identification using both sequencing of 16SrDNA and phenotypic methods was able to differentiate *E. fergusonii* from the other two bacteria in this study. Three *E. fergusonii* were carbapenem-resistant extended-spectrum beta-lactamase (ESBL) producers, showing resistance to imipenem, meropenem, ceftriaxone, ceftazidime and cefuroxime. Although none of the carbapenemases tested were identified, such isolates corresponded to the first carbapenem-resistant *E. fergusonii* recovered from human clinical samples. These isolates also carried *bla*_CTX-M_, *bla*_SHV_ or *bla*_TEM_ genes, and showed resistance to other antibiotics, such as fluoroquinolones, aminoglicosides or nitrofurantoin. This study has shown that *E. fergusonii* can be an unexplored reservoir of resistance to beta-lactams or other clinically relevant antibiotics.

Gonçalves Ribeiro et al., studied the occurrence of acquired AmpC β-lactamases (qAmpC) and characterized qAmpC-producing *Enterobacteriaceae* from different non-clinical (e.g., healthy humans, healthy livestock animals, food and aquatic sources) environments in Portugal [7]. The occurrence of qAmpC among *Enterobacteriaceae* from non-clinical origins was low, and compatible with the few available European studies. They corresponded to CMY-2-producing *Escherichia coli* from three humans (B22-ST4953; A0-ST665) and raw chicken meat (A1-ST48). CMY-2 genes were identified in non-typeable and IncA/C2 plasmids, the latter previously associated with CMY-2 genes in clinical strains from Portugal. The identification of CMY-2 in raw poultry meat highlighted the need of further monitorization in animals or at retail settings. Also, the indistinguishable Pulsed-Field-Gel-Eletrophoresis patterns identified between B2-ST4953 *E. coli* isolates recovered from two-family related individuals (husband and wife) indicated a common source for CMY-2 fecal carriage or human-to-human transmission. Most qAmpC studies from the literature have been focused particularly on clinical niches and/or specific bacterial species (*Escherichia coli*, non-typhoidal *Salmonella*), with this study being one of the few reports addressing the occurrence of qAmpC-producing *Enterobacteriaceae* among human, animal, and environmental settings.

Thummeepak et al., studied the copper phenotypes and genotypes of antibiotic-resistant *Acinetobacter baumanii* strains from clinical and environmental settings [8]. Whole genome sequencing revealed diverse copper tolerance genes among *A. baumanii*, showing that some of them (*cueR*, *pcoAB*, *oprC*) are intrinsic of this species, as they were present in all strains studied, while the acquired *copRS* embedded in a genomic island was only present in copper tolerant strains. By transcriptional assays the expression of those genotypes after copper induction incremented for intrinsic and acquired genes at micromolar and millimolar concentrations, respectively. The exposure of strains to copper also had an impact in the morphology and survival of a representative *A. baumanii* strain. Finally, this study showed that amikacin resistance and copper tolerance phenotypes were associated and that *bla*_NDM-1_ and the acquired *copRS* copper tolerance genotypes were statistically correlated. This work raises concerns about the efficacy of the use of copper in hospital and environmental settings in controlling the selection and spread of particular copper-tolerant *A. baumanii* strains.

Campos et al., provided us a comprehensive overview of the role of pig and pork meat in human salmonellosis at an international scale [9]. The authors highlighted the main factors that contribute to the persistence and dissemination of clinically relevant and pig-related serotypes and clones of *Salmonella*, one of the most frequent pathogens causing foodborne zoonosis worldwide. This review provides exhaustive and international data about: (a) the numbers of non-typhoidal *Salmonella* in pigs and pig production chain; (b) the worldwide epidemiology of major pig-related Salmonella serotypes (*Salmonella* Typhimurium, *Salmonella* 1,4, [5], 12:i:-, *Salmonella* Derby and *Salmonella* Rissen) often associated with human infections, including examples of spread of particular clones in different countries; (c) evidences of the emergence and spread of AMR pig/pork-related *Salmonella* isolates, including to the “Highest Priority Critically Important Antimicrobials” according to WHO; and (d) the potential contribution of metal tolerance genes (e.g., copper and silver) to the maintenance of pig-related serotypes in pig production setting, among other factors. The high incidence of resistance to clinically relevant antibiotics reported in diverse countries, together with the increasing demand for pork meat and the global trade in pig/pork products, reinforces the importance of a global integrated surveillance (“One Health”) approach, and the need for global mandatory interventions and strategies, including the improvement of biosecurity measures in farms during slaughtering/processing and at the retail/consumer level.
